# Evolutionary history and molecular epidemiology of rabbit haemorrhagic disease virus in the Iberian Peninsula and Western Europe

**DOI:** 10.1186/1471-2148-10-347

**Published:** 2010-11-10

**Authors:** Fernando Alda, Tania Gaitero, Mónica Suárez, Tomás Merchán, Gregorio Rocha, Ignacio Doadrio

**Affiliations:** 1 Dpto. Biodiversidad y Biología Evolutiva, Museo Nacional de Ciencias Naturales (CSIC). José Gutiérrez Abascal 2, 28006 Madrid, Spain; 2 Instituto de Investigación en Recursos Cinegéticos (CSIC-UCLM-JCCM). Ronda de Toledo s/n, 13071 Ciudad Real, Spain; 3 Dpto. Sanidad Animal, Facultad de Veterinaria, Universidad Complutense de Madrid. Avda. Puerta de Hierro s/n, 28040 Madrid, Spain; 4 Dpto. Ingeniería del Medio Agronómico y Forestal, Universidad de Extremadura. Av. Virgen del Puerto 2, 10600 Plasencia, Spain

## Abstract

**Background:**

Rabbit haemorrhagic disease virus (RHDV) is a highly virulent calicivirus, first described in domestic rabbits in China in 1984. RHDV appears to be a mutant form of a benign virus that existed in Europe long before the first outbreak. In the Iberian Peninsula, the first epidemic in 1988 severely reduced the populations of autochthonous European wild rabbit. To examine the evolutionary history of RHDV in the Iberian Peninsula, we collected virus samples from wild rabbits and sequenced a fragment of the capsid protein gene VP60. These data together with available sequences from other Western European countries, were analyzed following Bayesian Markov chain Monte Carlo methods to infer their phylogenetic relationships, evolutionary rates and demographic history.

**Results:**

Evolutionary relationships of RHDV revealed three main lineages with significant phylogeographic structure. All lineages seem to have emerged at a common period of time, between ~1875 and ~1976. The Iberian Peninsula showed evidences of genetic isolation, probably due to geographic barriers to gene flow, and was also the region with the youngest MRCA.

Overall, demographic analyses showed an initial increase and stabilization of the relative genetic diversity of RHDV, and a subsequent reduction in genetic diversity after the first epidemic breakout in 1984, which is compatible with a decline in effective population size.

**Conclusions:**

Results were consistent with the hypothesis that the current Iberian RHDV arose from a single infection between 1869 and 1955 (95% HPD), and rendered a temporal pattern of appearance and extinction of lineages. We propose that the rising positive selection pressure observed throughout the history of RHDV is likely mediated by the host immune system as a consequence of the genetic changes that rendered the virus virulent. Consequently, this relationship is suggested to condition RHDV demographic history.

## Background

Viruses containing RNA as their genetic material usually have a great capacity to adapt. This is so because the viral polymerases responsible for replicating the genome have a high error rate and therefore, many genomic variants are created at a high generation rate [1,2]. This high mutation rate of RNA viruses makes them excellent models for addressing evolutionary processes such as epidemic invasions, since their ecological and evolutionary dynamics occur at similar time scales [3-5].

RNA viruses are the causal agents of many emerging diseases and consequently the appearance, or reappearance of diseases caused by these viruses is not infrequent [2]. However, other ecological, social, health or behavioural factors besides their high rate of genetic variation can play an important role in the emergence of a disease [6].

Rabbit haemorrhagic disease (RHD) is a recent disease that was detected for the first time in 1984 in China, and attributed to rabbits (*Oryctolagus cuniculus*) imported from the former German Democratic Republic. The disease was described to cause sudden death without apparent deterioration of the rabbit's body condition, although haemorrhaging in the lungs was a frequent observation [7,8]. Hereafter, the disease rapidly spread to other Asian [9] and European [10] countries. The first report of RHD in Europe was in Italy in 1986 [11], and already in 1988 it was detected in wild rabbits in Spain [12] and in domestic rabbits in Russia, the Middle East, Africa, America and India [10].

The aetiological agent of RHD is a single stranded positive sense RNA virus belonging to the family Caliciviridae [13,14]. Since its discovery, many research efforts have been devoted to its study. However many issues remain unclear or controversial, such as its evolutionary origin or the causes of the rapid succession of epidemics in such a short period of time [15,16].

Several hypotheses have been proposed to explain the origins of rabbit haemorrhagic disease virus (RHDV), for example: (*i*) spread of brown hare syndrome virus - a closely related calicivirus - to the rabbit [17], (*ii*) changes in the properties of a non pathogenic virus that make it virulent; or (*iii*) the virus arose from a virus infecting another species [18]. The detection of RHDV specific antibodies and RNA fragments in rabbit serum samples from 1955 to 1980 [19-21], however, seem to support the idea that the virus was already circulating in Europe in an avirulent form before the first epidemic was detected in China in 1984 [16].

Several causes have also been proposed to explain the rapid expansion of RHD. Initially it was assumed that China was the origin from where the virus spread causing severe epidemics [18,22]. However, subsequent phylogenetic studies have demonstrated that the Chinese strains of the virus originated in Europe and currently circulating RHDV strains do not have a single origin. Rather, the virus seems to have originated at least twice in the past: firstly in Europe, without causing the disease, and later in the rabbits exported to China [16,23].

These studies have also detected a large number of RHDV evolutionary lineages that show low genetic divergence both within and among phylogenetic groups [17,19,24,25]. Some of these lineages do not present a clear geographic structure, but they do reflect a temporal structure whereby some lineages become extinct or less frequent, while others derived from them are able to persist and cause new outbreaks of the disease [16,17,24]. Despite showing no significant genetic differences in different geographic regions, RHDV does differ in its epidemiology and virulence [15,26-29].

Some regions, notwithstanding the drastic reductions suffered by rabbit populations after a first epidemic, have seen a decline in the initially high virulence of RHDV and this has enabled populations to gradually recover [15,26]. In the Iberian Peninsula, however, where rabbits are of great ecological and economic significance [30,31], RHD still affects many regions in which rabbit populations have been decimated or even extinguished [28,32].

This heterogeneity in the way RHDV affects rabbits in different geographic regions may indicate that there are factors such as the host, climate or population size that determine the epidemiology of the virus [15,28]. Regarding the host, in the Iberian Peninsula, the European rabbit is an autochthonous species. Analyses of mitochondrial and nuclear markers have revealed two highly divergent lineages in the European rabbit within the Iberian Peninsula that correspond to subspecies *O. c. algirus *and *O. c. cuniculus *[33-36]. These two lineages are the result of two divergent populations that evolved separately for a long time in two glacial refugia, one located in the southwest of the Iberian Peninsula and another in the northeast. A post-glacial expansion might have created a contact zone in the center of the Iberian Peninsula [37]. Furthermore, the northern *O. c. cuniculus *expanded outside of the Iberian Peninsula and originated all European rabbits, as well as those of North Africa, America, Australia, New Zealand and all the domestic breeds. This expansion originated an intense bottleneck effect that diminished considerably the genetic diversity in the *O. c. cuniculus *populations outside of the Iberian Peninsula [34,38-40]. Consequently, the rabbit in the Iberian Peninsula shows the largest genetic diversity across the world distribution of the species [34,41]. This genetic distinctiveness might pose different selective pressures and evolutionary constraints for RHDV compared to other regions. However, so far, comparative studies including the two rabbit lineages are scarce [16,42].

The objectives of the present study were: to examine the evolutionary history of RHDV in the Iberian Peninsula and to infer the virus' demographic history, with special focus on its Western European distribution. Given the genetic characteristics of this virus, we would expect to observe: (*i*) a high genetic diversity and (*ii*) a temporal genetic structure due to its high mutation rate. In terms of the possible origin of the virus and disease in the Iberian Peninsula, we could speculate that (*iii*) if a virulent form of the RHDV was introduced, the age of the most recent common ancestor should correspond to the time elapsed since the appearance of the disease, while if the virus already existed in an avirulent form, its age will be older. Also, according to the recent and rapid geographic expansion of RHDV we would expect (*iv*) its demographic history to reflect significant growth of its populations and that (*v*) if the intensity of the epidemics has decreased, or at least the number of hosts, a subsequent decline should also be observed.

## Methods

### Sampling and RNA isolation, amplification and sequencing

Rabbits were collected in Spain between 2003 and 2007 (Additional File 1). About 125 mg of lung tissue were homogenized in 1.25 ml of sterile PBS (8 mM Na_2_HPO_4_, 1.5 mM KH_2_PO_4_, 2.7 mM KCl, 137 mM NaCl, pH7.4) and centrifuged at 2500 × *g *for 15 min. The supernatant was collected and stored at -20°C upon RNA extraction.

Viral RNA was extracted from 100 μl of the lung homogenate using TriPure reagent (Roche) following the manufacturer's instructions. A fragment of the major capsid protein gene VP60 was amplified in all samples using the primers RHDV1, RHDV2, RHDV3 and RHDV4 [19].

The primers RHDV1 and RHDV4 were used to RT-PCR amplify a 698 bp fragment corresponding to positions 6096-6794 of the RHDV [43]. The RT-PCR reaction was conducted under the conditions indicated in the AccessQuick RT-PCR System kit (Promega) and the thermocycling program consisted of 45 min at 48°C for cDNA synthesis, followed by 2 min at 95°C, 40 cycles of 1 min at 95°C, 40 s at 64°C, 1 min at 72°C, and a final extension of 5 min at 72°C. Next, we performed a nested PCR with the primers RHDV2 and RHDV3 to amplify a 573 bp fragment corresponding to positions 6135-6719 of the RHDV. The PCR reaction contained 200 μM of dNTPs, 0.2 μM of each primer, 1 μg/μl of BSA, 1 U of *Taq *polymerase (Eppendorf), 2.5 μl of PCR buffer 10X (500 mM KCl, 100 mM Tris-HCl pH8.3, 15 mM Mg^2+^) and 0.5-2 μl of a 1:100 dilution of the RT-PCR product. The PCR program involved an initial denaturing step of 2 min at 95°C, 40 cycles of 1 min at 95°C, 1 min at 64°C and 1 min at 72°C, and a final extension step of 5 min at 72°C. All the RNA extraction and amplification processes were carried out in a laboratory specifically equipped for this purpose. All reactions included positive and negative controls. PCR products were purified using ExoSAP-IT (GE Healthcare) and sequenced using the BigDye Terminator v3.1 kit (Applied Biosystems) and the primers RHDV2 and RHDV3 in an automated sequencer ABI3730.

### Phylogenetic analysis

Chromatograms were checked visually and the consensus sequence was constructed for each sample using Sequencher 4.6 (Gene Codes Corporation). Nucleotide sequences were translated into amino acids using MacClade 4.05 [44] and manually aligned with other homologous sequences available in GenBank.

Recombination has been previously described in RHDV [45,46]. Although this seems to be a rather rare phenomenon that does not adversely affect rate estimations [16], we did not include sequences that had been identified as putative recombinants [16,45,46]. The alignment inferred from all of the available data (AD, *n *= 151, 563 bp) was subdivided in smaller data sets comprising sequences for the main geographic areas previously analyzed: Germany (GER), France (FRA1), United Kingdom (UK), China (CHI) and Iberian Peninsula (IB) (Additional File 2).

For a wider comparison, we included available data on non-overlapping VP60 sequences which could be of interest because of their temporal or geographic origins. Thus, an alignment of French RHDV isolates, consisting of non-overlapping sequences of 501 bp at the 3' end of the VP60 gene, was built (FRA2, *n *= 21) (Additional File 2) [24,47].

To evaluate the genetic variability of RHDV for each of these data sets from the main geographic areas we calculated their gene diversity (H*_d_*), nucleotide diversity (π) and number of polymorphic sites (*S*) using DnaSP 5.10.1 [48].

Phylogenetic relationships among all the RHDV strains (AD, *n *= 151) were inferred using different phylogenetic methods. As an outgroup we used four rabbit calicivirus (RCV) sequences (X96868, GQ166866, EU871528, NC011704), a seemingly avirulent, antigenically and genetically related to RHDV [49].

The evolutionary model that best fitted our data, according to the Akaike Information Criterion, was calculated in jModelTest 0.1.1 [50]. Firstly, we performed a Maximum Likelihood (ML) analysis, and used the best evolutionary method for our data as obtained in jModelTest 0.1.1, and allowed the program PhyML 3.0 [51] to optimize the tree topology and the values of gamma and proportions of invariant sites. Support for the ML trees was assessed by 1000 bootstrap replicates. Secondly, a Bayesian Inference (BI) analysis was performed using MrBayes 3.1.2 [52], simulating four simultaneous Markov chains (MCMC) for 4 × 10^6 ^generations each, and using a sampling frequency of 100 generations. The first 250,000 generations were discarded as burn-in. The BI analysis was performed considering one and three partitions of the data corresponding to the 1^st^, 2^nd ^and 3^rd ^codon positions each assigned different substitutions models. Bayesian posterior probabilities were obtained to assess the robustness of the BI trees.

Lastly, to test for the presence of statiscally significant geographical clustering in RHDV, we performed randomization tests on three tree-shaped statistics: the parsimony score (PS), the association index (AI) and the monophyletic clade statistic (MC) using BaTS 1.0 [53]. Randomizations were performed across the posterior distribution of trees obtained from MrBayes 3.1.2, hence accounting for phylogenetic uncertainty.

### Estimation of evolutionary rates, dates and past population demographic history

The overall population dynamics of RHDV (AD) was inferred in BEAST 1.5.3 [54] using the coalescent Bayesian Skyline Model [55] and an uncorrelated lognormal relaxed clock. Four independent analyses were performed under the best-fit substitution model and the SRD06 partition model, recommended for protein coding genes [54,56], and run for 5 × 10^7 ^generations and a sampling frequency of 1000 steps. All output generated were analyzed in Tracer 1.5 to test for convergence and mixing, and were used to estimate the substitution rate and time to the most recent common ancestor (tMRCA) of RHDV for each of the major lineages found and all the geographic regions.

We explored the changes through time of the relative genetic diversity (*N_e_*τ, where τ is the average generation time) of RHDV using the Bayesian Skyline Plot (BSP) method [55]. Median and 95% highest posterior density intervals (HPD) were obtained with Tracer 1.5.

We also tested for demographic changes using summary statistic based methods. The effective population size parameter (θ_0_) under a growth-decline population model (θ_1 _fixed at 1,000,000) was calculated for RHDV in DnaSP 5.10.1. Three time periods were defined based on the results obtained in the BSP analysis: avirulent period (before 1984), epidemic breakout (after 1984) and recent period (after 2000). Because samples sizes differed among time periods and, consequently, effective population size estimates might be biased, we constructed for the "after 1984" and "after 2000" periods 10 data sets with 9 random samples. These results were averaged and compared with the minimum sample size of RHDV before 1984.

### Selection analyses

To estimate selection pressures before and after the first epidemic of RHDV in 1984, we calculated the ratio between non-synonymous (*d_N_*) and synonymous (*d_S_*) substitutions for each individual codon using the fixed-effects likelihood (FEL) and random effects likelihood (REL) methods [57]. These analyses were performed in the online package Datamonkey [58]. Absence of recombination points was checked using GARD [59]. We established a value of *α *= 0.1 for FEL and BF = 20 for REL. In both cases, the *d_N_*/*d_S _*ratio was calculated using a neighbour-joining tree based on the best-fit evolutionary model.

As indicated above, to account for the effect of different sample sizes in the analysis, we analyzed 10 data sets with 9 random samples isolated after 1984, and compared with the results from RHDV isolated before the first epidemic.

## Results

RHDV was detected in 47 wild rabbits from Spain and it was possible to obtain a 563 bp sequence from each sample. All the sequences were analyzed unambiguously and we detected no evidence of infection by more than one viral strain in the same individual (GenBank accession numbers: HQ198325-HQ198371, Additional File 1).

Along with the homologous sequences available in GenBank, we constructed an alignment of 151 sequences (AD). From this alignment we extracted the data sets for Iberian Peninsula (IB, *n *= 71), France (FRA1, *n *= 8), United Kingdom (UK, *n *= 49), Germany (GER, *n *= 7) and China (CHI, *n *= 10). The alignment of non overlapping sequences from France (FRA2) contained 21 sequences isolated between 1988 and 2003 (Additional File 2).

The 151 RHDV sequences analyzed constituted 122 unique haplotypes (*H_d _*= 0.993 ± 0.003). The geographic regions that showed the highest genetic diversity were France and Germany. In contrast, among the 71 Iberian samples we found 45 haplotypes, which represented the lowest RHDV diversity (*H_d _*= 0.968 ± 0.010) (Additional File 3).

The best evolutionary model estimated by jModelTest 0.1.1 for our data was TrN+I+G. The gamma shape parameter was G = 0.574 and the proportion of invariable sites I = 0.172.

**Figure 1:**
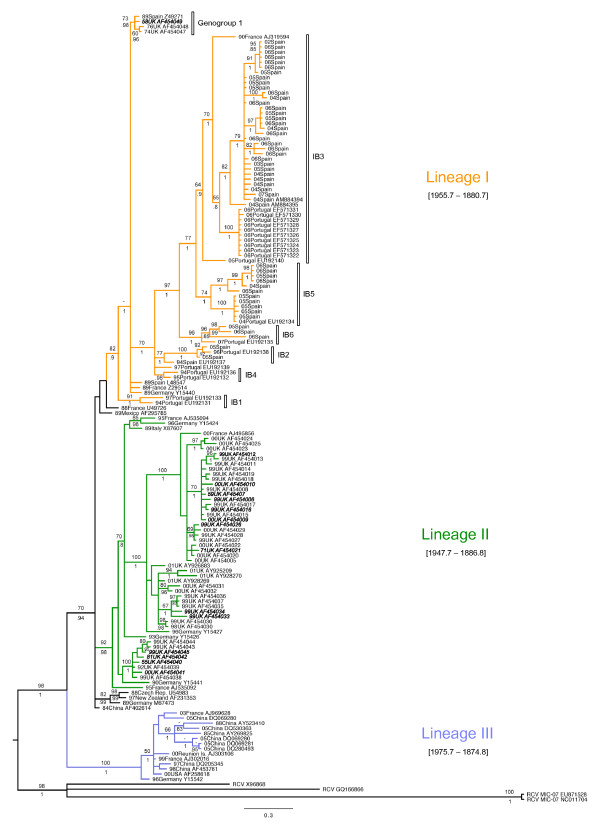
**Phylogenetic tree obtained by Bayesian inference for all the RHDV strains analyzed**. Numbers above branches indicate bootstrap values above 50 for ML analysis, and posterior probabilities above 0.80 for BI are shown below branches. Year and region of isolation is indicated for all samples. 95% HPD for the tMRCA of the main lineages found are indicated. Iberian clades within Lineage I are shown based on previous studies [24,42] and new data. Names in italics indicate avirulent RHDV strains.

The phylogenetic methods used rendered congruent topologies (Figure 1). Both analyses indicated three main lineages (Lineage I, II and III) in which most of the RHDV samples were included. Lineage I included RHDV strains isolated in different European regions (Figure 1), some of which had been isolated in the UK before the description of the disease in 1984 and others isolated during the first outbreaks in France, Germany and Spain (AST/89 and MC-89). Also, this lineage included all the Iberian RHDV samples isolated in Spain and Portugal from 1994 to 2007 (Figure 1). Six Iberian clades were described based on previous findings (Genogroup 1 [24], IB1, IB2 and IB3 [42]) and the new isolates from this study (IB4, IB5, IB6). No clear geographic structure was observed among the Iberian samples, although most of the clades were restricted in time, with the exception of IB3 that was the most widespread clade both in time and space (Figure 1, Additional File 1). In Lineage II, we exclusively found European strains of RHDV, mainly from the UK, Germany and France. Conversely, Lineage III included strains from different continents. German strains were found at a basal position with respect to all the Chinese strains, except a sample from 1984, which seemed more related to those of Lineage I and II, but without bootstrap or posterior probability support. This lineage also contained samples from the United States, France and Reunion Island in the Indian Ocean. Conversely, several RHDV strains from different regions (France, Germany, Czech Republic, China, Mexico and New Zealand) and strains prior to 1989 were not included in any of the three main lineages identified (Figure 1).

Overall, the test performed for the presence of phylogeographic structure using the PS and AI statistics, strongly rejected the hypothesis of panmixia (observed PS of 21.986, expected PS of 71.718 [*P *< 0.0001]; observed AI of 1.133, expected AI of 10.175 [*P *< 0.0001]). Furthermore, the MC statistic indicated that the correlation between phylogeny and taxa location was only significant for the samples from the Iberian Peninsula (observed MC_IB _of 41.046, expected MC_IB _of 3.681 [*P *= 0.01]), UK (observed MC_UK _of 21.040, expected MC_UK _of 2.757 [*P *= 0.01]) and China (observed MC_CHI _of 6.354, expected MC_CHI _of 1.175 [*P *= 0.01]), that appeared exclusively in Lineages I, II and III, respectively, with the exception of the "ancient" strains from the UK that were found in Lineage I. On the other hand, the RHDV isolates from France and Germany that appeared in all lineages, and in many cases were closely related to strains from other regions (Figure 1), did not show a significant geographic correlation.

The coalescence based Bayesian analysis implemented in BEAST 1.5.3 revealed that the MRCA for all the RHDV data dated back to ~1884 (95% HPD 1941.3-1730.7) and estimated an evolutionary rate of 5.48 × 10^-4 ^substitutions/site/year (95% HPD 2.79 × 10^-4^-8.10 × 10^-4^). The three main clades found showed similar 95% HPD intervals for their MRCA, from ~1875 to ~1976 (Figure 1), suggesting a close time of emergence for these lineages.

**Figure 2:**
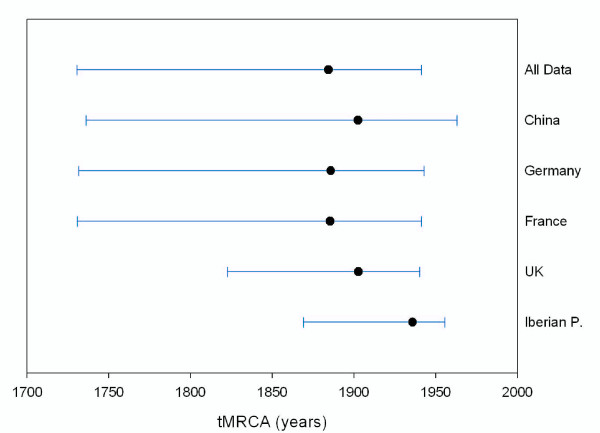
**Estimates of the tMRCA for the main geographical regions analyzed**. Dots indicate median tMRCA values and blue lines represent 95% HPD.

When the main geographic regions were considered, the youngest tMRCA was recovered for the Iberian RHDV (~1936, 95% HPD 1955.7-1869.2) followed by the isolates from the UK (~1898, 95% HPD 1940.3-1822.5). The remnant regions showed wider 95% HPD intervals that were similar to the age of the complete RHDV data set (Figure 2).

**Figure 3:**
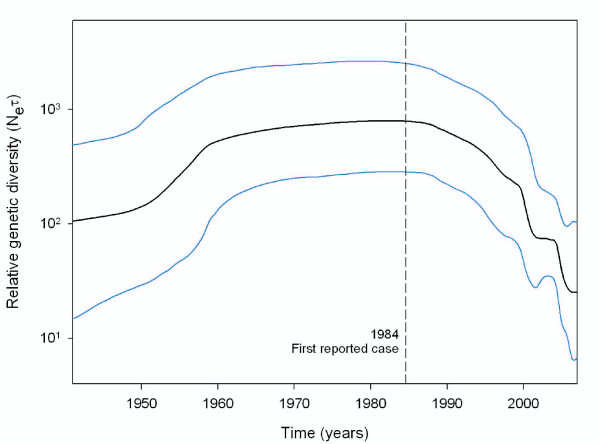
**Bayesian Skyline plot analysis of RHDV**. Graphical representation of relative genetic diversity (*N_e_*τ) changes across time of RHDV. Black line indicates the median and blue lines represent 95% HPD.

Bayesian Skyline Plot analysis of the demographic history of RHDV indicated that, as a whole, the relative genetic diversity of RHDV increased in the 1950's followed by a stationary phase until the mid 1980's. Thereafter, RHDV relative genetic diversity started to decline, especially in the late 1990's when this reduction was more drastic (Figure 3). Because samples were not balanced across years, the same analysis was performed removing those sequences dated before 1984 and presumably avirulent strains [15]. The plot recovered showed the same demographic and temporal pattern, although the stationary phase was not as clearly differentiated (data not shown).

The effective population size parameter θ_0 _estimated for the three main periods identified in the BSP also showed evidence of change in *N_e _*of RHDV across time. An increase of *N_e _*was observed between the avirulent period, before 1984 (θ_0 _= 11.597), and the samples after the first epidemic breakout, after 1984 (θ_0 _= 20.620 ± 2.407, average value based on 10 data sets of 9 random sequences). Subsequently, a reduction of *N_e _*was observed for the most recent isolates after 2000 (θ_0 _= 14.713 ± 5.812).

Adaptive selection analyses suggested an increase in the selective pressure of RHDV after the first epidemic breakout (Table 1). Before 1984 no codons were identified as positively selected by any method, and 29 codons were considered under negative selection by FEL, but not by the REL method. On the other hand, after 1984, codon 137 of the alignment was identified as positively selected considering all the data after 1984 and in 9 out of 10 random data sets analysed. Other codons (e.g. codon 155) were identified as positively selected, but not consistently among the random data sets. The FEL method also detected 100 negatively selected codons in all the samples after 1984 and 49.500 ± 5.126 codons under negative selection considering the 10 random data sets (Table 1).

**Table 1:** Adaptive selection analyses (FEL and REL) of RHDV performed in Datamonkey

	*d_N_/d_S_*	FEL		REL	
		**Positive selected**	**Negative selected**	**Positive selected**	**Negative selected**

**Before 1984**					
All data	0.173	0	29	0	0
**After 1984**					
All data	0.162	1	100	N/A	N/A
Average (10 data sets)	0.158 ± 0.017	1.600 ± 0.843	49.500 ± 5.126	2.400 ± 5.542	24.200 ± 28.389

## Discussion and Conclusion

### Genetic variability and origin of RHDV in the Iberian Peninsula

Although haplotype diversity was high and similar among regions, the nucleotide diversity of the RHDV strains varied among geographic regions. In the Iberian Peninsula, UK and China, nucleotide diversity was much lower than in France or Germany, and was equivalent to half the overall nucleotide diversity (Additional File 3). The low genetic diversity observed in these regions could suggest that the virus originated from a single introduction (e.g. as it is known to have occurred in China [7]), or that it is the outcome of geographic isolation, (e.g.the case of the British Isles). Thus, the genetic diversity of the virus in these regions could have been reduced as a consequence of a founder event in the former case, or of drift in the latter [60].

To date in the Iberian Peninsula, it was unknown if (*i*) RHDV was already circulating in an avirulent form, (*ii*) there was a single introduction of RHDV or (*iii*) subsequent contact with other strains occurred. The phylogenetic data obtained here indicate that all the field strains of RHDV in the Iberian Peninsula have a common ancestor and are closely related to strains AST/89 and MC-89 isolated during the first RHD outbreaks in Spain (Figure 1). The age of the MRCA of the Iberian strains is the youngest of all the regions analyzed (Figure 2), but still predates the first RHD outbreak in 1984 (~1936, 95% HPD: 1955.7-1869.2). Hence, it may be deduced that the virus introduced in the Iberian Peninsula came from a more ancient lineage already circulating in Europe. This fact was confirmed by the samples included in Lineage I together with AST/89 and MC-89, isolated in different regions of Europe since 1958 (Figure 1).

Dispersal of RHDV is fast and effective in short distances [61] because it is primarily transmitted by direct contact between sick animals or indirectly by dead animals or contaminated food [8]. However, long distances or geographic barriers, such as those existing in Great Britain or the Iberian Peninsula, can affect its dispersal [42]. The transport of rabbits or their products can also play an important role in passive dispersal of the virus [18]. According to the lack of any geographic structure of the Iberian strains (Additional File 1), it seems that movement of rabbits could have promoted RHDV dispersal within the Iberian Peninsula [32,62]. However, considering the significant global geographic association of all the Iberian strains, this is probably not the case for the import and export of rabbits abroad. Only one viral strain isolated in France in 2000 was found in the Iberian RHDV group (Figure 1) [42], which could have crossed the mountains transported by insects or the wind [63,64]. Therefore, the Pyrenees might also represent an effective barrier for RHDV, as it has been shown for many other organisms [65].

Owing to the rapid evolutionary rate of RNA viruses, their molecular phylogenies reveal both spatial and temporal patterns [4]. In France, six genetic groups have been described for RHDV that are mostly consistent with their dates of isolation but not their geographical location [24,47]. In the Iberian Peninsula, some clades spanned a wide range of time isolates, but most were restricted in time (Figure 1, Additional File 1). For example, clade IB1 was basal to all the other Iberian clades, including those strains isolated in 1989. Although this relationship was not supported by the ML analysis, it might indicate that, in the past, other related strains might have occurred (e.g. Genogroup 1, IB1, IB4) but are currently extinct. This temporal pattern, whereby lineages successively become extinct and others appear, is typical of RNA viruses [66,67], including calicivirus [68], and is mainly conditioned by positive selection [69].

### Phylogenetic relationships of RHDV

In general, the phylogenetic relationships resolved here are in line with the findings of studies that have analyzed different portions of the genome or geographic regions [15-17,19,23,24,42,47].

Three divergent groups were recovered from our phylogenetic reconstruction (Figure 1). These three groups (Lineages I, II and III) closely matched the lineages obtained in a phylogeny based on 43 complete sequences of the VP60 gene, in which two major lineages were described, one of which was divided in two well supported groups [45]. Thus, Lineages I, II and III described here would correspond to groups Ia, Ib and II respectively recovered in the phylogeny based on the complete VP60 gene sequence [45]. However, in other works in which partial sequences of the VP60 gene were examined, a much larger number of RHDV groups were defined, many of which are not supported [15,19,42,70,71].

As explained above, the genetic structure of RHDV has usually been described as being related to its year of isolation [17,19,24,41] and within temporal groups, a link to its place of origin emerges [41]. In our data, however, we observed a global geographical distribution of the lineages rather than a temporal structure of RHDV sequences (Figure 1).

The only lineage that did not contain samples before the first RHD outbreak was Lineage III, and we could consider that all the RHDV samples in this lineage arose from already virulent strains. On the other hand, Lineage I and II included either samples isolated before the first outbreak or avirulent strains. However, these lineages were not older than Lineage III. All lineages showed similar tMRCA estimates, suggesting a common time of emergence for all of them, between ~1875 and ~1976. This observed emergence pre-dates the documented emergence of RHD, a possibility that Kerr and colleagues had already suggested [16].

The RHDV strains from the Iberian Peninsula, UK and China appeared, almost exclusively, in Lineages I, II and III respectively. However, other strains such as those from France and Germany appeared in all the main clades (Figure 1). The presence of strains from different evolutionary lineages in the same region could be attributable to a greater extent to ancestral polymorphism and/or to higher flow of viruses in certain areas of central Europe [16], compared to more isolated regions such as the Iberian Peninsula and the British Isles. Furthermore, these isolated regions were those with the youngest tMRCA (Figure 2), suggesting that RHDV might have originated in continental Eurasia [16] and subsequently colonized the islands and southern peninsulas. However, different sampling sizes might affect the accuracy of these estimates, and conclusions should be drawn with caution.

### Demographic history and natural selection

The substitution rate estimated for the complete RHDV data set (5.48 × 10^-4 ^substitutions/site/year) was very similar to that reported recently for a similar data set (7.7 × 10^-4 ^substitutions/site/year, 95% HPD: 3.9 × 10^-4 ^- 11.0 × 10^-4 ^[16]). However, these rates were more than an order of magnitude lower than the rate estimated for the same gene region using a ML method (1.3 × 10^-3 ^substitutions/site/year, 0.59 × 10^-3 ^- 2.1 × 10^-3 ^[72]), and more than two orders of magnitude lower than the rate estimated for the capsid gene of other calicivirus [68]. Although the estimated substitution rate is within the range observed for single stranded RNA viruses, it more closely resembles the lower substitution rates recorded for viruses transmitted by arthropod vectors [72,73]. The relatively low substitution rate of this virus could therefore explain the long term persistence of certain strains in some populations many years after their introduction [74].

The BSP method, which allows the fitting of different demographic scenarios across time [55], distinguished three stages in the history of RHDV. First, a growth stage between 1950 and 1960; second, a stationary stage between 1960 and 1984 and finally a stage of decline beyond 1984 that became steeper at the end of the 1990s (Figure 3). The different demographic trends before and after the description of the first RHD outbreak match the different patterns of natural selection for each time range. Thus, during the growth and stationary stages, we detected no evidence of significant positive selection, whereas after 1984 evidence did emerge of positive selection, as well as an increase in the number of codons under negative selection over the VP60 fragment analyzed (Table 1).

In our data, codon 137, which is located in region E of the major capsid gene VP60, showed the strongest evidence of positive selection. The presence of positive selection in this region agrees with previous studies of RHDV and other calicivirus and with the fact that this region contains the main antigenic determinants [68,75]. Furthermore, the association between positive selection and antigenicity suggests that RHDV evolution is mainly driven by the host immune response [75].

Thus, the great virulence that characterized the first outbreaks of RHD would have caused a strong immune response in the rabbit, generating great selective pressure. In this way and as indicated by the selection analyses performed [75], there is ongoing selective pressure on certain RHDV strains, and consequently the fixation of favourable mutations or the purging of deleterious variants would result in the strong decrease in the relative genetic diversity recently observed for the virus [76,77].

## Authors' contributions

FA conceived the study, obtained and analyzed the molecular data and wrote the manuscript. TG and MS designed laboratory protocols and obtained molecular data. TM and GR collected samples and molecular data. ID provided funding and logistic support for the study. The final draft was read and approved by all the authors.

## Supplementary Material

Additional file 1 **MCC tree obtained in Beast for Lineage I and time span for each of the Iberian clades. Map and list of the Iberian samples analyzed in this study**. Click here for file

Additional file 2 **Origin and year of isolation of RHDV samples analyzed in this study**. Click here for file

Additional File 3 **Descriptive statistics of the genetic variability in RHDV**. Click here for file
