# Supplementary material for: Evolutionary history and molecular epidemiology of rabbit haemorrhagic disease virus in the Iberian Peninsula and Western Europe
Source: BMC Evol Biol. 2010 Nov 10;10:347. doi: 10.1186/1471-2148-10-347 (PMC2992527; doi:10.1186/1471-2148-10-347)
Supplement: Additional file 1 — MCC tree obtained in Beast for Lineage I and time span for each of the Iberian clades. Map and list of the Iberian samples analyzed in this study. [file 1471-2148-10-347-S1.PDF]

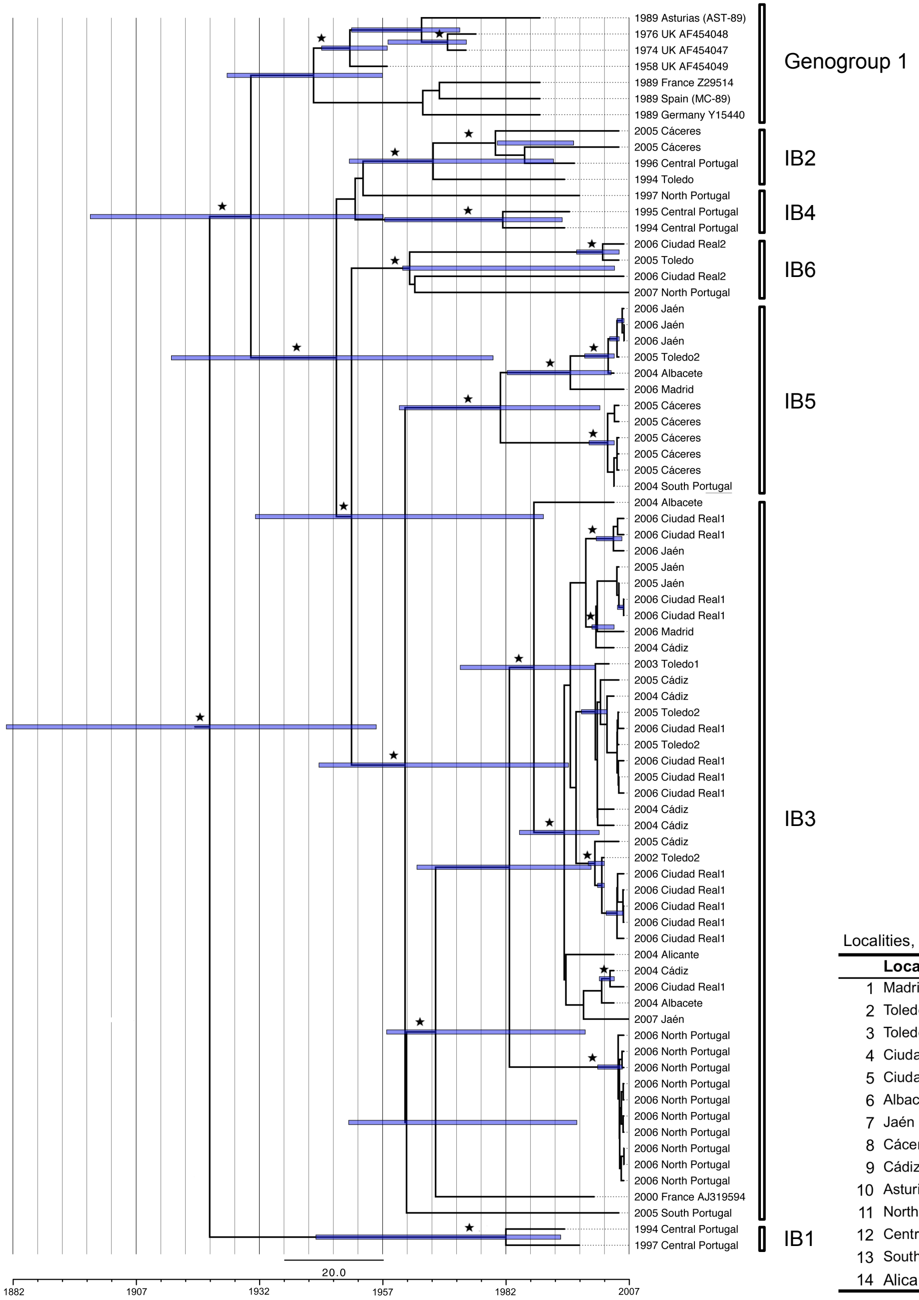

MCC tree obtained in BEAST for Lineage I. Bars indicate 95% HPD for the tMRCA of each clade. Stars denote posterior probabilities higher than 0.95.

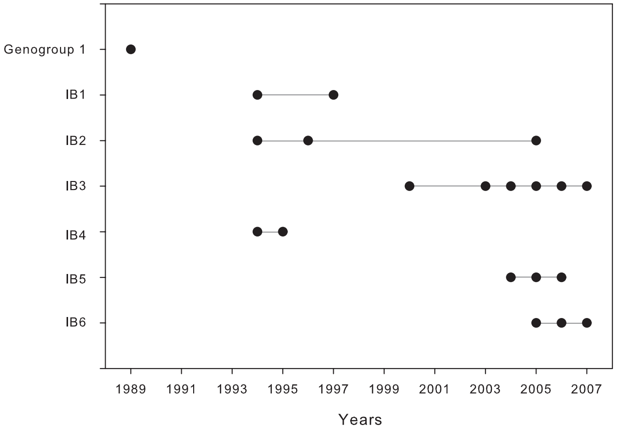

Time span for each of the Iberian clades identified in Lineage I.

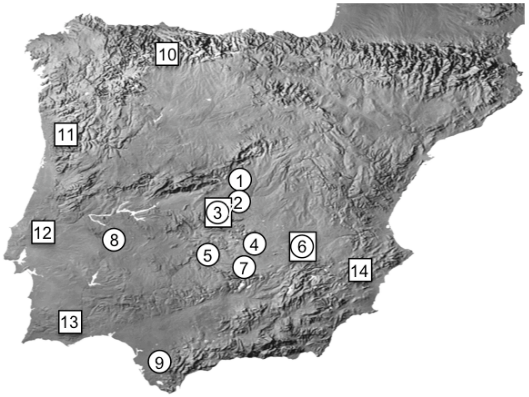

Map of RHDV sampling localities in the Iberian Peninsula. Circles represent samples isolated in this study and squares are samples obtained from the literature. Numbers correspond to localities listed in the table below.

Localities, number of samples, dates, host subspecies and GenBank accession numbers of the Iberian RHDV isolates used in this study.

	Locality	*n*	Dates	Host	GenBank
1	Madrid1	2	2006(2)	Contact zone *O. c. cuniculus* x *O. c. algirus*	HQ198334, HQ198353
2	Toledo1	3	1994(1), 2003(1), 2005(1)	Contact zone *O. c. cuniculus* x *O. c. algirus*	EU192137, HQ198333, HQ198354
3	Toledo2	6	2002(1), 2005(5)	Contact zone *O. c. cuniculus* x *O. c. algirus*	HQ198327-HQ198332
4	Ciudad Real1	13	2006(13)	Contact zone *O. c. cuniculus* x *O. c. algirus*	HQ198335-HQ198346, HQ198352
5	Ciudad Real2	2	2006(2)	*O. c. algirus*	HQ198362, HQ198363
6	Albacete	3	2004(3)	Contact zone *O. c. cuniculus* x *O. c. algirus*	AM884395
7	Jaén	7	2005(2), 2006(4), 2007(1)	*O. c. algirus*	HQ198325, HQ198326
8	Cáceres	7	2005(7)	*O. c. algirus*	HQ198365-HQ198371
9	Cádiz	6	2004(6)	*O. c. algirus*	HQ198355-HQ198358, HQ198360, HQ198361
10	Asturias	1	1989(1)	*O. c. cuniculus*	Z49271
11	North Portugal	12	1997(1), 2006(10), 2007(1)	*O. c. algirus*	EF571322-EF571331, EU192135, EU192139
12	Central Portugal	5	1994(2), 1995(1), 1996(1), 1997(1)	*O. c. algirus*	EU192131-EU192133, EU192136, EU192138
13	South Portugal	2	2004(1), 2005(1)	*O. c. algirus*	EU192134, EU192140
14	Alicante	1	2004(1)	Contact zone *O. c. cuniculus* x *O. c. algirus*	AM884394
