# Supplementary material for: Evolutionary history and molecular epidemiology of rabbit haemorrhagic disease virus in the Iberian Peninsula and Western Europe
Source: BMC Evol Biol. 2010 Nov 10;10:347. doi: 10.1186/1471-2148-10-347 (PMC2992527; doi:10.1186/1471-2148-10-347)
Supplement: Additional file 2 — Origin and year of isolation of RHDV samples analyzed in this study. [file 1471-2148-10-347-S2.PDF]

Additional File 2. Origin and year of isolation of RHDV samples analyzed in this study. The data set in which each sequence was included for the different analyses is indicated: AD: All data; IB: Iberian Peninsula (Spain and Portugal); UK: United Kingdom; FRA1: overlapping sequence for France; FRA2: non overlapping sequence for France; GER: Germany; CHI: China.

Origin	Date	Accession Number	Reference	Data set
Spain	1989	L48547	Viaplana & Villaverde 1996	AD, IB
Spain	1989	Z49271	Boga et al., 1994	AD, IB
Spain	1994	EU192137	Muller et al., 2008	AD, IB
Spain	2004	AM884395	Muller et al., 2008	AD, IB
Spain	2004	AM884394	Muller et al., 2008	AD, IB
Spain	2002	HQ198327	This study	AD, IB
Spain	2003	HQ198354	This study	AD, IB
Spain	2003	HQ198358	This study	AD, IB
Spain	2004	HQ198351	This study	AD, IB
Spain	2004	HQ198356	This study	AD, IB
Spain	2004	HQ198357	This study	AD, IB
Spain	2004	HQ198359	This study	AD, IB
Spain	2004	HQ198360	This study	AD, IB
Spain	2004	HQ198361	This study	AD, IB
Spain	2005	HQ198328	This study	AD, IB
Spain	2005	HQ198329	This study	AD, IB
Spain	2005	HQ198330	This study	AD, IB
Spain	2005	HQ198331	This study	AD, IB
Spain	2005	HQ198332	This study	AD, IB
Spain	2005	HQ198333	This study	AD, IB
Spain	2005	HQ198349	This study	AD, IB
Spain	2005	HQ198350	This study	AD, IB
Spain	2005	HQ198355	This study	AD, IB
Spain	2005	HQ198365	This study	AD, IB
Spain	2005	HQ198366	This study	AD, IB
Spain	2005	HQ198367	This study	AD, IB
Spain	2005	HQ198368	This study	AD, IB
Spain	2005	HQ198369	This study	AD, IB
Spain	2005	HQ198370	This study	AD, IB
Spain	2005	HQ198371	This study	AD, IB
Spain	2006	HQ198325	This study	AD, IB
Spain	2006	HQ198326	This study	AD, IB
Spain	2006	HQ198334	This study	AD, IB
Spain	2006	HQ198335	This study	AD, IB
Spain	2006	HQ198336	This study	AD, IB
Spain	2006	HQ198337	This study	AD, IB
Spain	2006	HQ198338	This study	AD, IB
Spain	2006	HQ198339	This study	AD, IB
Spain	2006	HQ198340	This study	AD, IB
Spain	2006	HQ198341	This study	AD, IB
Spain	2006	HQ198342	This study	AD, IB
Spain	2006	HQ198343	This study	AD, IB
Spain	2006	HQ198344	This study	AD, IB
Spain	2006	HQ198345	This study	AD, IB
Spain	2006	HQ198346	This study	AD, IB
Spain	2006	HQ198347	This study	AD, IB
Spain	2006	HQ198348	This study	AD, IB
Spain	2006	HQ198352	This study	AD, IB

Additional File 2. Continued.

Origin	Date	Accession Number	Reference	Data set
Spain	2006	HQ198362	This study	AD, IB
Spain	2006	HQ198363	This study	AD, IB
Spain	2007	HQ198364	This study	AD, IB
Portugal	1994	EU192136	Muller et al., 2008	AD, IB
Portugal	1994	EU192131	Muller et al., 2008	AD, IB
Portugal	1995	EU192132	Muller et al., 2008	AD, IB
Portugal	1996	EU192138	Muller et al., 2008	AD, IB
Portugal	1997	EU102139	Muller et al., 2008	AD, IB
Portugal	1997	EU192133	Muller et al., 2008	AD, IB
Portugal	2004	EU192134	Muller et al., 2008	AD, IB
Portugal	2005	EU192140	Muller et al., 2008	AD, IB
Portugal	2006	EF571322	Muller et al., 2008	AD, IB
Portugal	2006	EF571323	Muller et al., 2008	AD, IB
Portugal	2006	EF571324	Muller et al., 2008	AD, IB
Portugal	2006	EF571325	Muller et al., 2008	AD, IB
Portugal	2006	EF571326	Muller et al., 2008	AD, IB
Portugal	2006	EF571327	Muller et al., 2008	AD, IB
Portugal	2006	EF571328	Muller et al., 2008	AD, IB
Portugal	2006	EF571329	Muller et al., 2008	AD, IB
Portugal	2006	EF571330	Muller et al., 2008	AD, IB
Portugal	2006	EF571331	Muller et al., 2008	AD, IB
Portugal	2007	EU192135	Muller et al., 2008	AD, IB
United Kingdom	2000	AF454005	Moss et al., 2002	AD,UK
United Kingdom	1959	AF454007	Moss et al., 2002	AD,UK
United Kingdom	1999	AF454008	Moss et al., 2002	AD,UK
United Kingdom	1999	AF454009	Moss et al., 2002	AD,UK
United Kingdom	2000	AF454009	Moss et al., 2002	AD,UK
United Kingdom	2000	AF454010	Moss et al., 2002	AD,UK
United Kingdom	1999	AF454011	Moss et al., 2002	AD,UK
United Kingdom	1999	AF454012	Moss et al., 2002	AD,UK
United Kingdom	1999	AF454013	Moss et al., 2002	AD,UK
United Kingdom	1999	AF454014	Moss et al., 2002	AD,UK
United Kingdom	1999	AF454015	Moss et al., 2002	AD,UK
United Kingdom	1999	AF454016	Moss et al., 2002	AD,UK
United Kingdom	1999	AF454018	Moss et al., 2002	AD,UK
United Kingdom	1999	AF454019	Moss et al., 2002	AD,UK
United Kingdom	2000	AF454020	Moss et al., 2002	AD,UK
United Kingdom	1971	AF454021	Moss et al., 2002	AD,UK
United Kingdom	2000	AF454022	Moss et al., 2002	AD,UK
United Kingdom	2000	AF454023	Moss et al., 2002	AD,UK
United Kingdom	2000	AF454024	Moss et al., 2002	AD,UK
United Kingdom	2000	AF454025	Moss et al., 2002	AD,UK
United Kingdom	1999	AF454026	Moss et al., 2002	AD,UK
United Kingdom	1999	AF454027	Moss et al., 2002	AD,UK
United Kingdom	1999	AF454028	Moss et al., 2002	AD,UK
United Kingdom	2000	AF454029	Moss et al., 2002	AD,UK
United Kingdom	1999	AF454030	Moss et al., 2002	AD,UK
United Kingdom	1998	AF454030	Moss et al., 2002	AD,UK
United Kingdom	2001	AF454031	Moss et al., 2002	AD,UK
United Kingdom	2000	AF454031	Moss et al., 2002	AD,UK
United Kingdom	2000	AF454032	Moss et al., 2002	AD,UK

Additional File 2. Continued.

Origin	Date	Accession Number	Reference	Data set
United Kingdom	1999	AF454033	Moss et al., 2002	AD,UK
United Kingdom	1999	AF454034	Moss et al., 2002	AD,UK
United Kingdom	1999	AF454035	Moss et al., 2002	AD,UK
United Kingdom	1999	AF454036	Moss et al., 2002	AD,UK
United Kingdom	1999	AF454038	Moss et al., 2002	AD,UK
United Kingdom	1992	AF454039	Moss et al., 2002	AD,UK
United Kingdom	1955	AF454040	Moss et al., 2002	AD,UK
United Kingdom	2000	AF454041	Moss et al., 2002	AD,UK
United Kingdom	1981	AF454042	Moss et al., 2002	AD,UK
United Kingdom	1999	AF454043	Moss et al., 2002	AD,UK
United Kingdom	1999	AF454044	Moss et al., 2002	AD,UK
United Kingdom	1994	AF454045	Moss et al., 2002	AD,UK
United Kingdom	1974	AF454047	Moss et al., 2002	AD,UK
United Kingdom	1976	AF454048	Moss et al., 2002	AD,UK
United Kingdom	1958	AF454049	Moss et al., 2002	AD,UK
United Kingdom	1999	AF454006	Moss et al., 2003	AD,UK
United Kingdom	1999	AF454017	Moss et al., 2003	AD,UK
United Kingdom	1999	AF454046	Moss et al., 2004	AD,UK
Ireland	2001	AY925209	Forrester et al., 2006	AD,UK
Ireland	2001	AY926883	Forrester et al., 2006	AD,UK
Ireland	2001	AY928269	Forrester et al., 2006	AD,UK
Ireland	2001	AY928270	Forrester et al., 2006	AD,UK
France	1999	AJ302016	Le Gall-Recule et al., 2003	AD,FRA1,FRA2
France	2000	AJ319594	Le Gall-Recule et al., 2001 Unpublished	AD,FRA1,FRA2
France	2000	AJ495856	Le Gall-Recule et al., 2003	AD,FRA1,FRA2
France	1990	AJ535087	Le Gall-Recule et al., 2003	FRA2
France	1988	AJ535088	Le Gall-Recule et al., 2003	FRA2
France	1990	AJ535089	Le Gall-Recule et al., 2003	FRA2
France	1991	AJ535090	Le Gall-Recule et al., 2003	FRA2
France	1992	AJ535091	Le Gall-Recule et al., 2003	FRA2
France	1995	AJ535092	Le Gall-Recule et al., 2003	AD,FRA1,FRA2
France	1995	AJ535093	Le Gall-Recule et al., 2003	FRA2
France	1995	AJ535094	Le Gall-Recule et al., 2003	AD,FRA1,FRA2
France	1996	AJ535095	Le Gall-Recule et al., 2003	FRA2
France	1998	AJ535096	Le Gall-Recule et al., 2003	FRA2
France	1999	AJ535097	Le Gall-Recule et al., 2003	FRA2
France	1999	AJ535098	Le Gall-Recule et al., 2003	FRA2
France	2000	AJ535099	Le Gall-Recule et al., 2003	FRA2
France	1999	AJ535100	Le Gall-Recule et al., 2003	FRA2
France	1998	AJ535101	Le Gall-Recule et al., 2003	FRA2
France	1994	AJ535102	Le Gall-Recule et al., 2003	FRA2
France	2003	AJ969628	Le Gall-Recule et al., 2003 (Unpublished)	AD,FRA1,FRA2
France	2005	AM085133	Le Gall-Recule et al., 2005 (Unpublished)	FRA2
France	1988	U49726	Fischer et al., 1997	AD,FRA1
France	1989	Z29514	Rasschaert et al., 1995	AD,FRA1
Germany	1989	M67473	Meyers et al., 1991	AD,GER
Germany	1996	Y15424	Schirrmeier et al., 1997 (Unpublished)	AD,GER
Germany	1996	Y15427	Schirrmeier et al., 1997 (Unpublished)	AD,GER
Germany	1989	Y15440	Schirrmeier et al., 1997 (Unpublished)	AD,GER
Germany	1990	Y15441	Schirrmeier et al., 1997 (Unpublished)	AD,GER
Germany	1996	Y15542	Schirrmeier et al., 1997 (Unpublished)	AD,GER

Additional File 2. Continued.

Origin	Date	Accession Number	Reference	Data set
Germany	1996	Y15426	Schirrmeier et al., 1997 (Unpublished)	AD
Italy	1989	X87607	Rossi 1995 (Unpublished)	AD
Czech Rep.	1988	U54983	Gould et al., 1997	AD
China	1984	AF402614	Yan et al., 2001 (Unpublished)	AD
China	1998	AF453761	Liu et al., 2001 (Unpublished)	AD
China	1985	AY269825	Wang et al., 2003 (Unpublished)	AD
China	1988	AY523410	Xiang et al., 2005 (Unpublished)	AD
China	2005	DQ069280	Tian et al., 2007	AD
China	2005	DQ069281	Tian et al., 2007	AD
China	2005	DQ069282	Tian et al., 2007	AD
China	1997	DQ205345	Liu et al., 2005 (Unpublished)	AD
China	2005	DQ280493	Liu et al., 2005 (Unpublished)	AD
China	2005	DQ530363	Li et al., 2006 (Unpublished)	AD
USA	2000	AF258618	Neilan et al., 2000 (Unpublished)	AD
Mexico	1989	AF295785	Babcock et al., 2000 (Unpublished)	AD
New Zealand	1997	AF231353	Zheng et al., 2000 (Unpublished)	AD
Reunion Is.	2000	AJ303106	Le Gall-Recule et al., 2003	AD
