# Supplementary material for: Evolutionary history and molecular epidemiology of rabbit haemorrhagic disease virus in the Iberian Peninsula and Western Europe
Source: BMC Evol Biol. 2010 Nov 10;10:347. doi: 10.1186/1471-2148-10-347 (PMC2992527; doi:10.1186/1471-2148-10-347)
Supplement: Additional File 3 — Descriptive statistics of the genetic variability in RHDV. [file 1471-2148-10-347-S3.PDF]

Additional File 3. Descriptive statistics of the genetic variability in the RHDV. *n* = number of samples, bp = base pairs, *h* = number of haplotypes, H_d = gene diversity, *S* = number of polymorphic sites, π = nucleotide diversity, *SD* = standard deviation.

	n	*bp*	*h*	*Hd*	SD	*S*	π	SD
Iberian Peninsula (IBE)	71	563	45	0.968	0.010	150	0.048	0.003
France (FRA1)	8	548	8	1	0.063	112	0.078	0.083
France (FRA2)	21	497	21	1	0.015	129	0.061	0.005
United Kingdom (UK)	49	563	44	0.998	0.005	82	0.042	0.002
Germany (GER)	7	563	7	1	0.063	93	0.063	0.008
China (CHI)	10	563	9	0.978	0.003	82	0.042	0.009
All (AD)	151	563	122	0.993	0.003	228	0.086	0.002
